# Glyceraldehyde-3-phosphate dehydrogenase is largely unresponsive to low regulatory levels of hydrogen peroxide in *Saccharomyces cerevisiae*

**DOI:** 10.1186/1471-2091-11-49

**Published:** 2010-12-28

**Authors:** Luísa Cyrne, Fernando Antunes, Ana Sousa-Lopes, João Diaz-Bérrio, H Susana Marinho

**Affiliations:** 1 Centro de Química e Bioquímica, Faculdade de Ciências, Universidade de Lisboa, Campo Grande, 1749-016 Lisboa, Portugal; 2 Departamento de Química e Bioquímica, Faculdade de Ciências, Universidade de Lisboa, Campo Grande, 1749-016 Lisboa, Portugal

## Abstract

**Background:**

The reversible oxidation of protein SH groups has been considered to be the basis of redox regulation by which changes in hydrogen peroxide (H_2_O_2_) concentrations may control protein function. Several proteins become S-glutathionylated following exposure to H_2_O_2 _in a variety of cellular systems. In yeast, when using a high initial H_2_O_2 _dose, glyceraldehyde-3-phosphate dehydrogenase (GAPDH) was identified as the major target of S-glutathionylation which leads to reversible inactivation of the enzyme. GAPDH inactivation by H_2_O_2 _functions to reroute carbohydrate flux to produce NADPH. Here we report the effect of low regulatory H_2_O_2 _doses on GAPDH activity and expression in *Saccharomyces cerevisiae*.

**Results:**

A calibrated and controlled method of H_2_O_2 _delivery - the steady-state titration - in which cells are exposed to constant, low, and known H_2_O_2 _concentrations, was used in this study. This technique, contrary to the common bolus addition, allows determining which H_2_O_2 _concentrations trigger specific biological responses. This work shows that both in exponential- and stationary-phase cells, low regulatory H_2_O_2 _concentrations induce a large upregulation of catalase, a fingerprint of the cellular oxidative stress response, but GAPDH oxidation and the ensuing activity decrease are only observed at death-inducing high H_2_O_2 _doses. GAPDH activity is constant upon incubation with sub-lethal H_2_O_2 _doses, but in stationary-phase cells there is a differential response in the expression of the three GAPDH isoenzymes: Tdh1p is strongly upregulated while Tdh2p/Tdh3p are slightly downregulated.

**Conclusions:**

In yeast GAPDH activity is largely unresponsive to low to moderate H_2_O_2 _doses. This points to a scenario where (a) cellular redoxins efficiently cope with levels of GAPDH oxidation induced by a vast range of sub-lethal H_2_O_2 _concentrations, (b) inactivation of GAPDH cannot be considered a sensitive biomarker of H_2_O_2_-induced oxidation in vivo. Since GAPDH inactivation only occurs at cell death-inducing high H_2_O_2 _doses, GAPDH-dependent rerouting of carbohydrate flux is probably important merely in pathophysiological situations. This work highlights the importance of studying H_2_O_2_-induced oxidative stress using concentrations closer to the physiological for determining the importance of protein oxidation phenomena in the regulation of cellular metabolism.

## Background

The preferential and reversible oxidation of specific cysteine residues present in enzymes, transcription factors and receptors has been proposed to be the major mechanism by which oxidants may integrate into cellular signal transduction pathways [1,2]. The sulfhydryl (SH) group of cysteine residues, especially when present in an environment that decreases its pKa, can be oxidized by hydrogen peroxide (H_2_O_2_), the main cellular reactive oxygen species. The major product of the reaction between a protein cysteinyl thiol and hydrogen peroxide is a protein sulfenic acid [3,4] that, unless in a shielded environment, is a transient intermediate that undergoes a range of secondary reactions [1,2]. The protein sulfenic acid can form (a) mixed disulfides with low-molecular weight thiols, mainly glutathione (S-glutathionylation), (b) intramolecular disulfides when vicinal thiols are present, (c) intermolecular disulfides between proteins or (d) reversible condensation with an adjacent amide to form a sulfenylamide. All these oxidations are reversible and, therefore, provide a mechanism by which protein function may be controlled by changes in cellular H_2_O_2 _concentration. When the levels of oxidant exposure are higher further oxidation of cysteinyl sulfenic acids can occur, leading to the formation of cysteinyl sulfinic and sulfonic acids [1,2], which is considered largely irreversible *in vivo *[5]. Moreover, these higher levels of oxidative stress may often result in excessive disulfide bonding, and in the misfolding, aggregation, and degradation of proteins leading, eventually, to cell death [6,7].

Glyceraldehyde-3-phosphate dehydrogenase (GAPDH) is a classic glycolytic enzyme that is active as a tetramer of identical 37 kDa subunits catalyzing the oxidative phosphorylation of glyceraldehyde-3-phosphate to 1,3-diphosphoglycerate by converting NAD^+ ^to NADH. More recently, GAPDH emerged as a multifunctional protein with defined functions in numerous subcellular processes, namely a primary role in apoptosis and in a variety of critical nuclear pathways [8,9]. In the yeast *Saccharomyces cerevisiae *(*S. cerevisiae*) three related but not identical GAPDH enzymes with different specific activities are encoded by unlinked genes designated *TDH1*, *TDH2 *and *TDH3 *[10]. None of the *TDH *genes are individually essential for cell viability, but a functional copy of either *TDH2 *or *TDH3 *is required since *tdh2*Δ*tdh3*Δ cells are not viable [11].

Studies with mammalian cells have identified GAPDH as a target of oxidative modifications resulting in decreased activity following exposure to H_2_O_2 _[12,13]. GAPDH has an active-site cysteine residue which, following exposure to H_2_O_2_, can be oxidized to an intramolecular disulfide and cysteic acid [14] and also undergo S-glutathionylation [13]. In *S. cerevisiae *growing in exponential phase, GAPDH was also identified as a major target of S-glutathionylation [15,16] and also carbonylation [17-19] and a sharp decrease in its enzymatic activity was observed [15,16,18,20] following exposure to H_2_O_2_. In cell extracts exposed to H_2_O_2 _both Thdh2p and Thdh3p are S-glutathionylated, but in vivo only S-glutathionylation of Thd3p is observed [15,16,20].

Studies of GAPDH inactivation and S-glutathionylation in *S. cerevisiae *cells [15-18,20] have been performed in the exponential phase of growth using bolus additions of high doses of H_2_O_2 _that cause high levels of cell death, and so it is difficult to assess the possible regulatory role of H_2_O_2 _on GAPDH activity by inducing reversible GAPDH thiol oxidation, including S-glutathionylation. Therefore, our main aim with this study was to establish in *S. cerevisiae *cells at what levels, in the range of low to moderate H_2_O_2 _concentrations, is H_2_O_2 _able to cause an inhibition of GAPDH activity due to reversible and irreversible oxidation of the enzyme. To achieve this, instead of the typical H_2_O_2 _bolus addition, we exposed cells to controlled steady-state levels of H_2_O_2_, mimicking the physiological production of H_2_O_2 _in vivo. This methodology allows to study the biological effects of low to moderate levels of H_2_O_2 _[21] and by using it we were able to show that in yeast the overall GAPDH activity only decreases at high doses of H_2_O_2_. We also characterized the differential expression of the three Tdh isoenzymes when cells were exposed to H_2_O_2 _under our controlled conditions of H_2_O_2 _delivery. In stationary-phase cells Tdh1p expression emerged as a target for regulation by low H_2_O_2 _levels.

## Results

### What are regulatory H_2_O_2 _concentrations?

A high enough H_2_O_2 _dose will eventually trigger the oxidation of cysteine residues, and so it is important to define whether the doses at which H_2_O_2 _is observed to inhibit GAPDH activity are either in the regulatory low range, or in the toxic high range of H_2_O_2 _concentrations.

**Figure 1:**
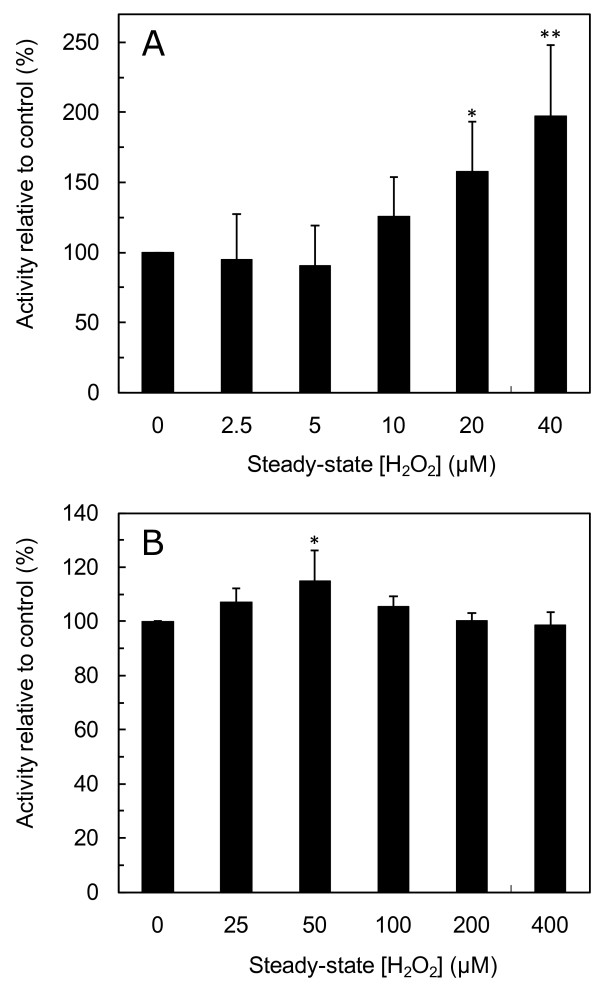
**Low concentrations of H_2_O_2 _increase catalase activity in *S. cerevisiae***. Exponential-phase cells (A) and stationary-phase cells (B) were exposed to the indicated steady-state H_2_O_2 _concentrations for 60 min. The values are the mean ± standard deviation of n≥5 independent experiments. The activity of control cells (without added H_2_O_2_) was 0.29 ± 0.07 min^-1^mg^-1 ^for exponential-phase cells and 4.51 ± 0.67 min^-1^mg^-1 ^for stationary-phase cells. *P < 0.05 vs control; **P < 0.001 vs control.

To define what a low to moderate steady-state H_2_O_2 _dose is, the activity of catalase, an important antioxidant enzyme that removes H_2_O_2_, was measured. In exponential phase cells, catalase activity was upregulated for a 20 μM steady-state H_2_O_2 _exposure and for 40 μM H_2_O_2 _there was a two-fold increase in catalase activity (Figure 1A). As to stationary-phase, cells treated with H_2_O_2 _on the lower range of concentrations studied (50 μM) showed an increase in catalase activity (Figure 1B). This increase in catalase activity was modest when compared to that observed in exponential-phase cells, probably because catalase activity in stationary-phase cells is 15-fold higher than in exponential-phase cells, reflecting a probable higher formation of H_2_O_2 _due to mitochondrial respiration. However, for our purpose this increase in catalase activity observed at 50 μM steady-state H_2_O_2 _was sufficient to identify a range of concentrations where H_2_O_2 _triggers a regulatory response. *S. cerevisiae *has two catalases, peroxisomal catalase (catalase A) and cytosolic catalase (catalase T), encoded by the *CTA1 *and *CTT1 *genes respectively, and both are known to be induced by H_2_O_2 _[22]. H_2_O_2_-dependent upregulation of *CTT1 *expression has been shown to be dependent on the transcription factors Yap1p, Skn7p [23] and Msn2p/Msn4p [24] and it should be pointed out that the minimum H_2_O_2 _concentration needed to activate those transcription factors, when using a bolus addition, was found to be 100 μM [25,26].

**Figure 2:**
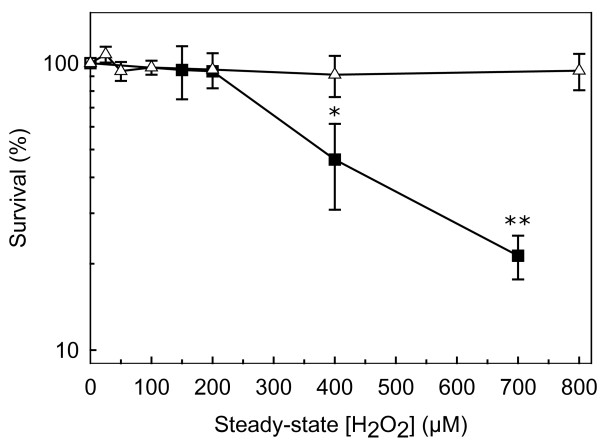
**Susceptibility of *S. cerevisiae *cells to steady-state H_2_O_2_-induced oxidative stress**. Survival fractions are shown for exponential-phase cells (■) and stationary-phase cells (△) that were exposed to steady-state H_2_O_2 _between 25 μM and 800 μM for 60 min. The values are the mean ± standard deviation of n≥3 independent experiments. *P < 0.05 vs control; **P < 0.01 vs control.

To define what a high dose of H_2_O_2 _is, the survival of *S. cerevisiae *wild-type cells exposed to steady-state concentrations of H_2_O_2 _was determined. For exponential-phase cells, under our conditions, the value of 200 μM can be defined as the border between moderate and high toxic conditions because higher steady-state H_2_O_2 _concentrations lead to significant cell death (approximately 55% for 400 μM H_2_O_2 _and 80% for 700 μM H_2_O_2_) (Figure 2). For stationary-phase cells, doses up to 800 μM may be considered moderate because they did not alter cell survival, which is in agreement with the known higher resistance of these cells to H_2_O_2 _when compared to exponential-phase cells [27].

In conclusion, steady-state H_2_O_2 _concentrations in the range 20 μM to 50 μM may be viewed as triggering a regulatory H_2_O_2 _response in *S. cerevisiae*.

### Low to moderate concentrations of H_2_O_2 _do not lead to changes in GAPDH activity in *S. cerevisiae *exponential-phase cells

**Figure 3:**
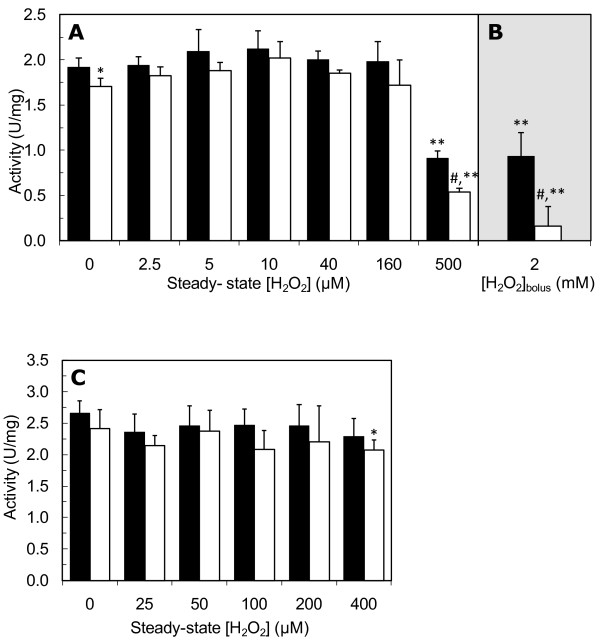
**Low H_2_O_2 _concentrations do not inactivate GAPDH in *S. cerevisiae***. GAPDH activity in exponential-phase cells (A, B) or stationary-phase cells (C) treated with either the indicated steady-state H_2_O_2 _concentrations for 60 min or (B) with a 2 mM H_2_O_2 _bolus addition for 30 min was measured in the presence (closed bars) and in the absence of DTT (dashed bars) in the assay medium. The difference of the activity measured in the presence and in the absence of DTT is a measure of reversible GAPDH thiol oxidation. The values are the mean ± standard deviation of 3≤n < 10 independent experiments. *P < 0.05 vs DTT; ^#^P < 0.01 vs DTT; **P < 0.001 vs control.

GAPDH activity was determined with and without the reducing agent DTT in the assay medium. The difference in the enzyme activity in both assays measures GAPDH reversible inactivation due either to S-glutathionylation, formation of a sulphenic acid or of an intramolecular disulfide. In control cells the activity of GAPDH measured in the absence of DTT was approximately 10% lower than in the presence of DTT (Figure 3A), indicating either that the enzyme was partially oxidized in vivo or that it became oxidized during protein extraction. This partial oxidation of GAPDH in unstressed cells has been previously found [28,29]. If the reversible inactivation of GAPDH induced by H_2_O_2_, namely by S-glutathionylation, has an important role in the regulation of GAPDH activity during oxidative stress it should be expected that a higher reversible inactivation of the enzyme would occur when cells are exposed to low to average H_2_O_2 _concentrations (i.e., 20-50 μM, as defined in the previous point).

When exponential-phase cells were exposed to increasing steady-state H_2_O_2 _concentrations no changes in GAPDH activity determined both in the presence and absence DTT were observed for H_2_O_2 _concentrations up to 160 μM (Figure 3A). This dose is 8 times higher than the H_2_O_2 _concentration identified as being able to induce catalase activity and is close to the steady-state 150 μM H_2_O_2 _dose that induces a multi-factorial cellular response causing adaptation to H_2_O_2 _[30]. Previous studies of GAPDH inactivation by H_2_O_2 _in yeast were done with bolus additions [15,16,18,20], which require the use of a much higher initial H_2_O_2 _concentration. When a typical bolus addition of 2 mM H_2_O_2 _was added to exponential-phase cells (Figure 3B), after 30 min GAPDH activity had decreased to 10% of that in control cells (1.73 ± 0.10 and 0.17 ± 0.09 U/mg in control and H_2_O_2_-treated cells respectively). Part of this inactivation induced by the H_2_O_2 _bolus addition was due to reversible GAPDH thiol oxidation since GAPDH activity in H_2_O_2_-treated cells measured in the presence of DTT was about 47% of that in control cells (1.99 ± 0.18 and 0.93 ± 0.20 U/mg for control cells and H_2_O_2_-treated cells respectively). So, a much higher steady-state concentration of H_2_O_2 _(500 μM) was used in order to try to obtain a similar GAPDH inactivation to that observed when using a bolus addition of H_2_O_2_. When exponential-phase cells were exposed to a steady-state 500 μM H_2_O_2 _concentration, GAPDH activity showed a marked decrease (to 26% and 40% of GAPDH activity in the absence and presence of DTT, respectively) (Figure 3A). However, it should be noted that similarly to what happens with a 2 mM bolus H_2_O_2 _addition [16], cell survival when cells are subjected to a steady-state 500 μM H_2_O_2 _concentration is lower than 50% (Figure 2).

In conclusion, for cells in the exponential phase GAPDH activity is largely unresponsive to low to moderate H_2_O_2 _levels, in opposition to a widely accepted view. GAPDH activity does decrease, but only for H_2_O_2 _concentrations that cause significant cell death.

### H_2_O_2 _does not inactivate irreversibly GAPDH in *S. cerevisiae *stationary-phase cells

GAPDH activity in stationary-phase cells is about 25% higher than in exponential-phase cells (2.71 ± 0.20 U/mg and 2.03 ± 0.17 U/mg respectively, P < 0.001). No changes in GAPDH activity were found for stationary-phase cells treated with steady-state H_2_O_2 _concentrations up to 400 μM (Figure 2C) both in the presence and absence of DTT, when compared with control cells. However, for cells exposed to 400 μM H_2_O_2 _there was a significant difference in GAPDH activity determined in the presence and absence of DTT, indicating a small increase in the GAPDH reversible oxidation induced by H_2_O_2_. Nevertheless, this H_2_O_2 _concentration is much higher than the 50 μM, identified in Figure 1 which induces catalase. Therefore, GAPDH may not be considered to be a sensitive target of H_2_O_2 _in stationary phase cells.

### Tdh2p/Tdh3p expression is not altered by H_2_O_2 _in exponential-phase *S. cerevisiae *cells

**Figure 4:**
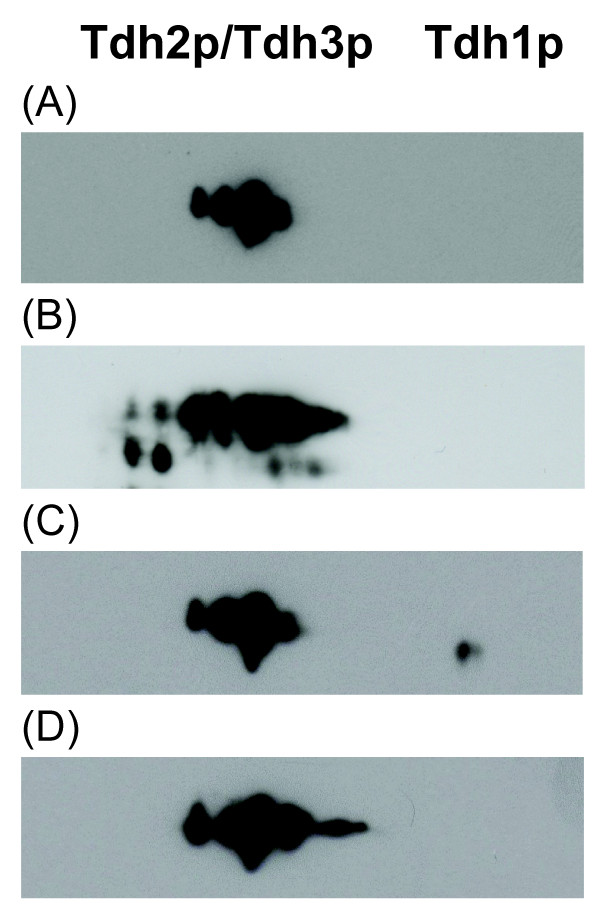
**Characterization of the antibodies used for GAPDH detection**. Representative (n≥3) two-dimensional analysis of Tdh1p, Tdh2p and Tdh3p (only the region with the 3 GAPDH isoenzymes is shown) *S. cerevisiae *cells with immunodetection made by Western blot as described in materials and methods are shown. (A) wt stationary-phase cells with identification using mouse anti-GAPDH (Chemicon MAB374). Identification using rabbit anti-GAPDH was performed in (B) wt exponential-phase cells, (C) wt stationary-phase cells, and (D) *tdh1*Δ stationary-phase cells.

**Figure 5:**
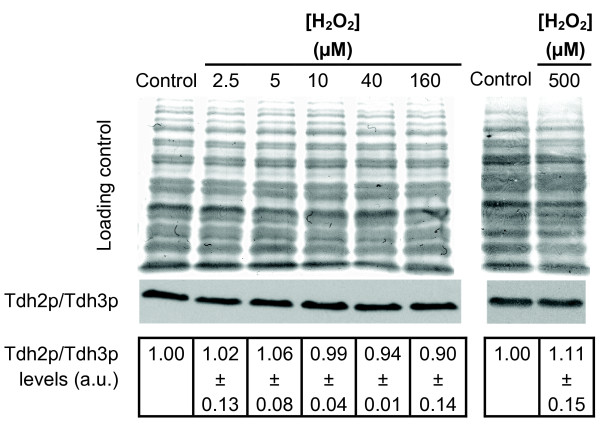
**Tdh2p and Tdh3p protein levels are not affected by H_2_O_2 _in exponential-phase *S. cerevisiae *cells**. Cells were treated with the steady-state H_2_O_2 _concentrations indicated for 60 min. Representative protein loading and immunoblot analysis of Tdh2p/Tdh3p (n = 5) and signal intensity quantification expressed as the mean ± standard deviation in arbitrary units (a.u.) relative to control. Mouse anti-GAPDH that only reacts with Tdh2p and Tdh3p was used for Tdh2p/Tdh3p identification.

**Figure 6:**
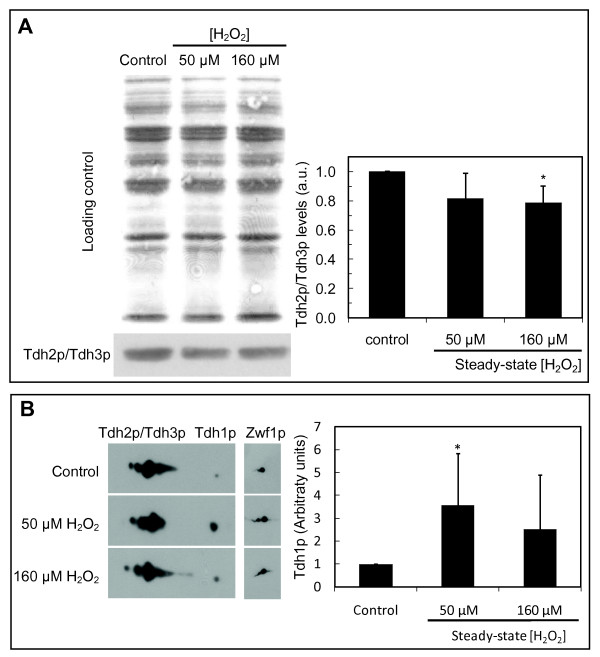
**Low concentrations of H_2_O_2 _upregulate Tdh1p and high concentrations downregulate Tdh2p and Tdh3p protein levels in stationary-phase *S. cerevisiae *cells**. Cells were treated with the steady-state H_2_O_2 _concentrations indicated for 60 min. (A) Representative protein loading and immunoblot analysis of Tdh2p/Tdh3p (n = 6) and signal intensity quantification expressed as the mean ± standard deviation in arbitrary units (a.u.) relative to control. Mouse anti-GAPDH, which only reacts with Tdh2p and Tdh3p, was used for Tdh2p/Tdh3p identification (see Figure 3). (B) Representative two-dimensional analysis of Tdh1p, Tdh2p and Tdh3p (only the region with the three GAPDH isoenzymes is shown, n≥4) in stationary-phase cells with immunodetection made by Western blot using rabbit anti-GAPDH as described in materials and methods; signal intensity quantification of GAPDH levels normalized to Zwf1p, which was used as internal control, and expressed as the mean ± standard deviation in arbitrary units (a.u.) relative to control is also shown. *P < 0.05 vs control.

Up until now we have focused on the activity of GAPDH as a whole. Next we evaluate the pattern of expression of each of the three GAPDH isoenzymes. To this end, 2-dimensional gel electrophoresis followed by immune detection was performed (Figures 4, 5 and 6). Previous studies have shown that in two-dimensional gels Tdh2p and Tdh3p slightly overlap, while Tdh1p migrates in a different position [18,31]. As can be seen in Figure 4 the mouse anti-GAPDH (Chemicon MAB374) reacted only with Tdh2p and Tdh3p (Figure 4A), while the rabbit anti-GAPDH reacted with all three isoforms present in stationary-phase cells (Figure 4C). Tdh1p identification was confirmed using a *tdh1*Δ strain (Figure 4D). Tdh1p was not detected in exponential-phase cells (Figure 4B), which is in agreement with previous studies [18,32,33] where Tdh1p has been described as a minor GAPDH isoenzyme only expressed in the stationary phase of growth. Tdh1p was also not detected when exponential-phase cells were exposed to 20 μM and 160 μM steady-state H_2_O_2 _(results not shown). Since Tdh2p and Tdh3p levels were difficult to obtain from the 2D-analysis, Western blot analysis from SDS-PAGE using the mouse anti-GAPDH followed by signal intensity analysis was used to quantify both Tdh2p and Tdh3p levels when cells were exposed to H_2_O_2_. In Figure 5 it can be seen that exposure of exponential-phase cells up to 500 μM H_2_O_2 _did not alter the levels of Tdh2p/Tdh3p expression. This contradicts observations obtained with higher H_2_O_2 _doses, where a downregulation of Tdh2p/Tdh3p *de novo *synthesis [34] and Tdh2p/Tdh3p mRNA steady-state levels [35] was observed, and highlights the multitude of possible responses caused by different H_2_O_2 _doses.

### H_2_O_2 _upregulates Tdh1p expression and downregulates Tdh2p/Tdh3p expression in stationary-phase *S. cerevisiae *cells

Unlike what happens in exponential-phase cells where exposure to H_2_O_2 _does not alter GAPDH expression, in stationary-phase cells a slight downregulation of Tdh2p/Tdh3p expression was observed for higher H_2_O_2 _concentrations (Figure 6A). In fact, after exposure to 160 μM H_2_O_2 _Tdh2p/Tdh3p steady-state levels were about 80% of those in control cells. On the other hand, Tdh1p expression in stationary-phase cells was upregulated by H_2_O_2 _since Tdh1p levels were 3.5 fold higher in cells exposed to steady-state 50 μM H_2_O_2 _than in control cells (Figure 6B). Therefore, Tdh1p emerges as a protein that is strongly induced by low concentrations of H_2_O_2 _in stationary-phase cells.

## Discussion

It has been established for a number of years that oxidation of particular proteins containing reactive cysteine residues, such as GAPDH, occurs when mammalian and yeast cells are exposed to H_2_O_2_. However, in most of these studies, cells are exposed to exogenous H_2_O_2 _concentrations several orders of magnitude higher (millimolar) than the physiological concentration [36], due to its rapid removal by intracellular catalases and peroxidases. Therefore, protein oxidation and enzyme inactivation caused by H_2_O_2 _has been mostly established for situations when cells were under severe oxidative stress and already dying either by apoptosis or necrosis, and thus very far from a regulatory situation. Much less is known [e.g. [37,38]] about the effect of H_2_O_2 _concentration ranges (micromolar) where H_2_O_2 _may affect signaling processes and protein function through reversible changes in protein thiols redox state. In this work, a calibrated method of H_2_O_2 _delivery to cells was used where cells were exposed to steady-state concentrations of H_2_O_2 _throughout the experiment, which allows the use of much smaller concentrations of H_2_O_2 _[21,39]. This made possible to study for the first time in *S. cerevisiae *cells how low concentrations of H_2_O_2 _affect GAPDH activity and expression. Our study showed that GADPH activity in *S. cerevisiae *cells, both in the exponential-phase and stationary-phase of growth, is not inhibited by exposure to exogenous low to moderate H_2_O_2 _levels. This was unexpected considering previous studies of GAPDH inactivation by H_2_O_2 _in yeast [15-18,20] and suggests that in yeast cells exposed to low to moderate H_2_O_2 _concentrations the enzymatic systems responsible for the maintenance of cellular protein thiols [20,40], have enough activity to maintain the SH groups of the reactive cysteine residues in the GAPDH active centre mostly in the reduced form, thus avoiding inactivation of the enzyme. Also, *S. cerevisiae *exponential-phase cells response to high H_2_O_2 _doses includes the upregulation of the proteins involved in the glutaredoxin and thioredoxin systems [34,41], which are responsible for the reduction of protein disulfides.

Stationary-phase cells have higher resistance to H_2_O_2 _than exponential phase cells which is in part due to the higher catalase activity (Figure 2) and also to a lower cellular permeability to H_2_O_2 _[27]. So, when exposed to the same exogenous H_2_O_2 _concentration stationary-phase cells have a lower intracellular H_2_O_2 _concentration than exponential-phase cells [27]. Also, the levels of glutaredoxins [41] and thioredoxins [42] expression are upregulated in the stationary phase of growth. This probably partially explains why in stationary-phase cells overall GAPDH activity is not inhibited for the whole range of H_2_O_2 _concentrations used in this work. Unlike what happens for exponential-phase cells, where Tdh2p and Tdh3p levels are not altered by exposure to H_2_O_2 _and Tdh1p is not detected, in stationary-phase cells Tdh2p and Tdh3p levels were slightly downregulated at higher H_2_O_2 _concentrations. In spite of this result, overall GAPDH activity and total GAPDH mRNA steady-state levels in stationary-phase cells are not altered by H_2_O_2 _(results not shown). A possible explanation for this discrepancy is that, in spite of the decrease in the total amount of Tdh3p plus Tdh2p, the relative proportion of Tdh2p versus Tdh3p is increased. It is known that the three isoenzymes have different kinetic constants since it has been determined that Tdh3p has a two- to three-fold lower apparent V_max _than Tdh2p [11]. Also, it can be hypothesized that Tdh1p upregulation observed at low H_2_O_2 _concentrations is compensating for the decreased levels of Tdh2p/Tdh3p, the overall net result being that GAPDH activity is maintained constant.

Tdh1p levels have also been previously found to be induced by reductive stress caused by an excess of cytoplasmic NADH in an anaerobically grown *gpd2*Δ strain lacking one of the glycerol-3-phosphate dehydrogenases [43]. This upregulation of Tdh1p expression by changes in cellular redox state may be related to its function in signaling pathways, possibly the Hog1p pathway. In fact, in *Schizosaccharomyces pombe*, a recent study [44] showed that in response to H_2_O_2 _stress, the Cys-152 of Tdh1p is transiently oxidized. This oxidation enhances the association of Tdh1p with Mcs4p (mitotic catastrophe suppressor) and Mpr1p, a protein that transfers a phosphoryl group from the Mak2/Mak3 sensor histidine kinases activated by H_2_O_2_. The equivalent pathway in *S. cerevisiae *is the Hog1p pathway involved in the response to increased extracellular osmolarity, which is also activated by oxidative stress [45,46].

In the last years the paradigm found in the literature is that GAPDH is a sensitive protein target of H_2_O_2 _and that its inhibition by H_2_O_2 _is a controlled response advantageous for cells because it diverts glucose from the glycolytic pathway to the pentose phosphate pathway, thus increasing the availability of NADPH for antioxidant enzymes [12,15,47]. In favor of such an hypothesis, a recent study [48] found that H_2_O_2_-induced GAPDH inactivation to about 20% of control resulted in an increase in the levels of the metabolites of the pentose phosphate pathway. Also, a mathematical model indicated that for those levels of GAPDH inhibition there was a change in the NADPH/NADP^+ ^ratio from 6.5 to 19, with a steep increase in the ratio for levels of GAPDH inactivation greater than about 40%. Ralser *et al*. [48] used that evidence as support of a model where GAPDH functions as a cellular switch that reroutes the carbohydrate flux to maintain the cytoplasmic NADPH/NADP^+ ^equilibrium to counteract oxidative stress. However, as shown by this work, those levels of GAPDH inactivation can only be attained under severe oxidative stress when cells are dying. Therefore, this rerouting of the glycolytic carbohydrate flux due to GAPDH inhibition, although it may be important in pathophysiological situations, is not a physiological regulatory response. Moreover, the higher GAPDH activity observed after exhaustion of carbon sources in stationary-phase cells when compared to glucose-based growth in exponential-phase cells argues against the rerouting of carbohydrate flux being an important physiological mechanism. This higher GAPDH activity in stationary-phase cells also supports the paradigm [8,9] that GAPDH has other functions in the cell besides its role in glycolysis.

## Conclusions

In conclusion, this work has shown that in *S. cerevisiae *GAPDH activity is largely unresponsive to low to moderate doses of H_2_O_2_. This highlights the importance of studies of H_2_O_2_-induced oxidative stress using concentrations closer to the physiological for determining the importance of protein oxidation phenomena in the regulation of cellular metabolism. Most other studies of GAPDH in yeast have used considerably higher concentrations of H_2_O_2 _than we used which lead to conclusions that are possibly only important in situations of necrosis and apoptosis. The notable exception to the lack of GAPDH response to low H_2_O_2 _concentrations is the H_2_O_2_-induced upregulation of Tdh1p expression in stationary-phase *S. cerevisiae *cells.

## Methods

### Compounds, antibodies and other materials

Yeast extract, bactopeptone, yea < st nitrogen base and agar were from Difco, Detroit, MI, USA. Glucose oxidase (*Aspergillus niger*) and digitonin were from Aldrich, Steinheim, Germany. D, L-glyceraldehyde-3-phoshate, L-α-amino acid oxidase (*Crotalus atrox*), bovine liver catalase and phenylmethylsulfonyl fluoride were from Sigma Chemical Company, St. Louis, MO, USA. Hydrogen peroxide was obtained from Merck & Co., Inc., Whitehouse Station, NJ, USA. Anti-glyceraldehyde-3-phoshate dehydrogenase monoclonal antibody was from Chemicon International Inc., Temecula, CA, USA and rabbit anti-glyceraldehyde-3-phoshate dehydrogenase antibody was a kind gift from Dr. Pedro Moradas-Ferreira, Instituto de Biologia Molecular e Celular, Porto, Portugal. Anti-mouse IgG-horseradish peroxidase conjugate antibody (sc-2005) and anti-rabbit IgG-horseradish peroxidase conjugate antibody (sc-2004) were obtained from Santa Cruz Biotechnology, Inc., Santa Cruz, CA, USA).

### Yeast strains, media and growth conditions

The *S. cerevisiae *strains used in this work, BY4741 (*MATa; his3Δ1; leu2Δ0; met15Δ0; ura3Δ0*) and *tdh1*Δ (isogenic to BY4741 with *YJL052w::kanMX4*), were obtained from EUROSCARF, Frankfurt, Germany. For all experiments *S. cerevisiae *cells were inoculated at an OD_600 _of 0.05 and cultured in synthetic complete (SC) medium containing 6.8% (w/v) yeast nitrogen base, 2% (w/v) glucose, and amino acids as indicated in [49], at 30°C and with shaking at 160 rpm. Exponential-phase cells were harvested at 0.5 OD_600 _(~2 × 10^7 ^cells) and stationary-phase cells were harvested after 7 days.

### Exposure to H_2_O_2 _and cell survival

Exponential-phase cells were exposed to steady-state H_2_O_2 _concentrations during 60 min in fresh SC medium at 30°C with shaking at 160 rpm using glucose oxidase [30]. For stationary-phase cells, cells were resuspended in an amino acid solution (0.1 M phosphate buffer, pH 7.4, containing amino acids with the same concentrations as in SC medium) and L-α-amino acid oxidase was used for H_2_O_2 _generation [27]. Briefly, an aliquot containing the desired H_2_O_2 _concentration was added to the cells together with either glucose oxidase or L-α-amino acid oxidase at such an activity that the rapid consumption of the added initial H_2_O_2 _concentration by the cells was compensated, thus keeping H_2_O_2 _concentration constant (steady-state) during the assay. Cell survival after exposure to steady-state concentrations of H_2_O_2 _was monitored by plating diluted sample aliquots on YPD plates [50] and counting colonies after 48 h.

### Protein extracts

Crude protein extracts were prepared by glass bead lysis. Cells were suspended either in 100 mM potassium phosphate buffer pH 7.4 containing 1 mM PMSF or, in the case of two-dimensional gel electrophoresis analysis, in Tris HCl 10 mM buffer pH 8.0 containing 2 μM DTT, protease inhibitors: 1 mM PMSF, 1.5 μg/ml benzamidine, 10 μg/ml leupeptin, and 1 μg/ml pepstatin, added to an equal volume of glass beads, and vortexed for 7 cycles of 1 min of vortexing followed by 1 min of cooling on ice. The mixture was then centrifuged at 8000 × *g*, 20 min at 8°C. The supernatants were used for determinations of enzyme activities, total protein [51] and GAPDH levels by Western blot after SDS-PAGE or two-dimensional gel electrophoresis.

### Enzyme activities

Glyceraldehyde-3-phosphate dehydrogenase activity in protein extracts was assayed according to [52], with some minor modifications. NADH formation was measured at 340 nm (ε = 6220 M^-1^cm^-1^) during 4 min at 25°C in 1 ml reaction medium with 0.015 M sodium pyrophosphate buffer pH 8.5 containing 0.03 M sodium arsenate, 0.26 mM NAD^+^, 3.5 mM dithiothreitol (DTT) and protein extract. The reaction was started by the addition of 0.525 mM DL-glyceraldehyde-3-phosphate. The activity assay was also performed in the absence of DTT. One unit is defined as the quantity of enzyme reducing 1 μmol NAD^+^/min at 25°C and pH 8.5.

In exponential-phase cells catalase activity was determined in situ. Cell membrane permeabilization was achieved by incubating cells with 0.02% (w/v) digitonin, dissolved in dimethylsulphoxide, in 0.1 M potassium phosphate buffer, pH 6.5, for 15 min at 30°C with shaking. H_2_O_2 _consumption (100 μM initial concentration) by permeabilized cells suspended in the permeabilization buffer at 30°C was followed using an oxygen electrode (Hansatech Instruments Ltd, Norfolk, England). In stationary-phase cells catalase activity was measured spectrophotometrically in protein extracts by following H_2_O_2 _consumption (initial concentration, 10 mM) at 240 nm at 25°C for 2 min according to Aebi [53]. H_2_O_2 _concentrations were plotted on a semi-logarithmic graph against time and the first order rate constant (catalase activity) was calculated.

### SDS-PAGE and two-dimensional gel electrophoresis

SDS-PAGE was carried out according to [54]. 50 μg of total proteins were denaturated and analyzed in 7.5% (w/v) polyacrylamide (w/v). For two-dimensional analysis overnight precipitation using acetone (1 ml) was done to protein extracts (150 μg total protein). The pellet was resuspended in 250 μl of rehydration solution [8 M urea, 0.5% (w/v) CHAPS, 0.2% (w/v) DTT, 0.5% (v/v) IPG buffer pH 3-10, 0.002% (w/v) bromophenol blue], were loaded into the strip holders (Amersham Biosciences) and IEF (first dimension) was carried out on linear IPGs (pH 3-10; 13 cm long IPG) and achieved using the Ettan IPGphor system (GE Healthcare). Strips were rehydrated with 150 μg of proteins extracts for 12 h at room temperature. Strips were then focused according to the following electrical conditions at 20°C: 150 V for 1 h, 300 V for 1 h, 600 V for 1 h, from 600 to 4000 V in 90 min and 4000 V for 4 h. After focusing IPG strips were placed for 15 min in equilibration buffer [6 M urea, 30% (v/v) glycerol, 2% (w/v) SDS, 0.05 M Tris-HCl, pH 8.8, 0.002% (w/v) bromophenol blue] containing 1% (w/v) DTT and, subsequently, for 15 min in the same buffer but replacing the 1% (w/v) DTT by 2.5% (w/v) iodoacetamide [54]. The second dimension was carried out on 10% polyacrylamide gradient gels (16 × 14 × 0.15 cm) at 55 V per gel and 21°C until the dye front reached the bottom of the gel, according to Laemmli [54]. Identification of Tdh1p, Tdh2p and Tdh3p in protein extracts from wt and *tdh1*Δ cells after SDS-PAGE or two-dimensional gel electrophoresis was made by Western blot as described below.

### Western blot analysis

For immunodetection, the proteins were transferred to nitrocellulose membrane (0.45 nm; Schleicher and Schuell) in a semi-dry system, using 39 mM glycine, 48 mM Tris, 0.0375% (w/v) SDS and 20% (v/v) methanol pH 9.2 as transfer buffer [55], during 60 min at 0.8 mA/cm^2^, not exceeding 25 V. Blots were blocked at room temperature for 1 h in PBS (4.3 mM Na_2_HPO_4_, 1.4 mM KH_2_PO_4_, 137 mM NaCl and 2.0 mM KCl, pH 7.4) containing 5% (w/v) fat-free milk powder, before incubation with the primary antibody. The mouse anti-GAPDH (Chemicon MAB374) was used with a dilution of 1:1000 whereas the rabbit anti-GAPDH was used with a dilution of 1:5000, followed by incubation for 1 h. After washing 5 times in PBS containing 0.1% (v/v) Tween-20, the blots were incubated for 1 h with the secondary antibody conjugated to horseradish peroxidase (sc-2005; 1:2000 or sc-2004; 1:5000), washed extensively with PBS and detected by enhanced chemiluminescence (ECL kit, Amersham). Protein levels were quantified by signal intensity analysis by using *ImageJ *[56], normalized to the protein loading, either membrane stained with Ponceau S when doing SDS-PAGE, or Zwf1p levels, and expressed as arbitrary units relative to control. For stationary-phase cells Zwf1p was used as internal control since its expression is not altered by the H_2_O_2 _concentrations used in this study (Cyrne *et al*., unpublished).

### Statistical analysis

Results presented are the means ± standard deviation of the indicated independent experiments. To determine statistical significance among groups data were analyzed using one-way analysis of variance (ANOVA) followed by post analysis with the Tukey-Kramer multiple comparisons test or, alternatively, when appropriate, Student's t-test.

## List of abbreviations

DTT: dithiothreitol; GAPDH: Glyceraldehyde-3-phosphate dehydrogenase; *S. Cerevisiae*: *Saccharomyces cerevisiae*; SC medium: synthetic complete medium;

## Authors' contributions

LC, FA and HSM conceived the study, and participated in its design and coordination. LC performed the 2D-electrophoresis and western blot analysis. FA performed the steady-state H_2_O_2 _exposure of cells and helped to draft the manuscript. ASL, JDB and HSM performed the enzyme activity assays. HSM analyzed and interpreted the data and drafted the manuscript. All authors read and approved the final manuscript.
